# Isolation of a *Gordonia rubripertincta* bacteriophage, BluerMoon, from topsoil in Lubbock, Texas

**DOI:** 10.1128/mra.00725-25

**Published:** 2025-11-24

**Authors:** Laurissa N. Miller, Natalie Block, Whitney Dickens, Carson Bellew, Christian Deluna, Francesca Makilan, Malli Bhakta, Ashleigh Crawford, Trinity Criner, Chase Drucker, Aqsa Fayyaz, Jasmine Goh, Caitlyn Guetersloh, Claire Jansen, Dana Pham, Andrea Resendez, Austen Rowell, Fahareen B. Mosharraf, Allie C. Smith, Lisa M. Bono

**Affiliations:** 1 Department of Biological Sciences, Texas Tech University 6177, Lubbock, Texas, USA; 2 Teaching, Learning, and Professional Development Center, University Library, Texas Tech University 6177, Lubbock, Texas, USA; 3 TrUE Scholars Program Center for Transformative Undergraduate Experiences, Texas Tech University 6177, Lubbock, Texas, USA; 4 Honors College, Texas Tech University 6177, Lubbock, Texas, USA; Portland State University, Portland, Oregon, USA

**Keywords:** *Gordonia* spp., *Gordonia rubripertincta*, environmental bacteriophage, genome sequencing, genome analysis, SEA-PHAGES, phage isolation, genome assembly

## Abstract

We isolated, annotated, and analyzed the genome of an environmental bacteriophage. BluerMoon was discovered from topsoil in Lubbock, Texas, as part of the Science Education Alliance–Phage Hunters Advancing Genomics and Evolutionary Sciences (SEA-PHAGES) program. BluerMoon is a member of the DJ cluster according to the Actinobacteriophage database, PhagesDB (https://phagesdb.org).

## ANNOUNCEMENT

*Gordonia rubripertincta* is a gram-positive bacterium, part of phylum Actinomycetota (1), commonly found in soil and has bioremediation potential (2). Through the Science Education Alliance–Phage Hunters Advancing Genomics and Evolutionary Sciences (SEA-PHAGES) program, *G. rubripertincta* was used as the host bacteria to isolate, characterize, sequence, and annotate a bacteriophage from soil samples collected in Lubbock, TX (3).

BluerMoon was collected from a sample of soil at a depth between 1 and 5 cm on 30 August 2023 at coordinates 33.58482,-101.87825. The sample was suspended in peptone yeast calcium (PYCa) broth and centrifuged at 2,000 × *g* (3,500 rpm) for 10 min. Debris was filtered out using a 0.22-µm syringe filter, and 500 µL of the supernatant was incorporated into a liquid culture of *G. rubripertincta* NRRL B-16540. Enriched isolation samples were incubated at 30°C at 220 rpm for 3 days and then diluted and plated using an overlay technique (4). A clear plaque was picked and suspended in a solution of 400 µL of PYCa broth and 400 µL of 80% glycerol. Then, 10 µL of this mixture was looped into 3 mL of PYCa. From this, 1 µL, 10 µL, or 100 µL were overlayed with 250 µL of host and 3 mL of molten top agar to make lacy (webbed) plates using an overlay technique for high-titer lysates (>1 × 10^9^ pfu/mL) (4). DNA of the sample was processed using a Quantabio Extracta Plus DNA kit (Cat. # 95213-050). A BioTek Take3 plate reader was used to measure the DNA concentration. The Illumina DNA Prep tagmentation kit was used to prepare the whole genome for sequencing on an Illumina NextSeq 2000 platform using a 300-cycle flow cell kit generating 2 × 150 bp paired reads.

Host sequence contamination was removed using Bowtie2 v2.5.3 using a closely related host sequence (accession number CP136136.1) (5). Read quality was checked using Fastp v0.23.4 (6). SPAdes genome assembler v3.15.5 was used for genome assembly (7). Depth and coverage were determined using BEDTools v2.31.0 and SAMtools v1.6 (8, 9). Genome annotation was completed using DNA Master v5.23.3 and Phage Evidence Collection and Annotation Network (PECAAN) (10, 11). Glimmer v3.0 and GeneMark v2.5 were used to manually validate and predict the genes during annotation (12). BLAST v2.11.01 (13) (using PhagesDB.org and NCBI non-redundant databases), Phamerator (14) (using Actino_draft database v578), NCBI Conserved Domains databases (15), HHPRED v3.0 (16) (using the PDB_mmCIF70), and Pfam-v36.0 (17) were utilized to predict gene function. All software applications mentioned were used with default configurations (Table 1).

**TABLE 1:** Characterization of the reported bacteriophages

Parameter	Data for the isolated bacteriophage BluerMoon
Genome length (bp)	61,118
Sequencing coverage (x)	2,424
No. of predicted genes	90
No. of tRNAs	0
No. of hypothetical proteins of unknown function	65
GC content (%)	68.8%
Facility performing sequencing	SeqCoast Genomics
GenBank accession no.	PV251866
SRA accession no.	SRR31925873
BioProject accession no.	PRJNA488469

BluerMoon was isolated from an enrichment culture on *G. rubripertincta*. BluerMoon is part of the DJ cluster within the Actinobacteriophage database, PhagesDB (https://phagesdb.org), and is lytic. DJ cluster phage has an average GC content of 51.5%, an average genome size of 60,476 bp, and infects *Gordonia* spp. We manually classified BluerMoon as having a 3’ sticky overhang genome termini as previously described (18). The lack of tRNAs found within BluerMoon was determined using tRNAscan-SE v2.0 and ARAGORN v1.2.38, consistent with all phages in the DJ cluster (19, 20).

## Data Availability

This project has been deposited in GenBank under accession number PV251866 and the NCBI Sequence Read Archive database (SRA) under accession number SRR31925873. The BioProject number for BluerMoon is PRJNA488469.
